# Cytotoxic Effects of Fascaplysin against Small Cell Lung Cancer Cell Lines

**DOI:** 10.3390/md12031377

**Published:** 2014-03-07

**Authors:** Gerhard Hamilton

**Affiliations:** 1 Ludwig Boltzmann Cluster of Translational Oncology, A-1090, Vienna, Austria; E-Mail: gerhard.hamilton@toc.lbg.ac.at; Tel./Fax: +43-1-40400-6627; 2 Department of Surgery, Medical University of Vienna, A-1090 Vienna, Austria

**Keywords:** fascaplysin, cyclin-dependent kinase, small cell lung cancer, cytotoxicity, reactive oxygen species, camptothecin, poly(ADP-ribose)-polymerase inhibitor

## Abstract

Fascaplysin, the natural product of a marine sponge, exhibits anticancer activity against a broad range of tumor cells, presumably through interaction with DNA, and/or as a highly selective cyclin-dependent kinase 4 (CDK4) inhibitor. In this study, cytotoxic activity of fascaplysin against a panel of small cell lung cancer (SCLC) cell lines and putative synergism with chemotherapeutics was investigated. SCLC responds to first-line chemotherapy with platinum-based drugs/etoposide, but relapses early with topotecan remaining as the single approved therapeutic agent. Fascaplysin was found to show high cytotoxicity against SCLC cells and to induce cell cycle arrest in G1/0 at lower and S-phase at higher concentrations, respectively. The compound generated reactive oxygen species (ROS) and induced apoptotic cell death in the chemoresistant NCI-H417 SCLC cell line. Furthermore, fascaplysin revealed marked synergism with the topoisomerase I-directed camptothecin and 10-hydroxy-camptothecin. The Poly(ADP-ribose)-Polymerase 1 (PARP1) inhibitor BYK 204165 antagonized the cytotoxic activity of fascaplysin, pointing to the involvement of DNA repair in response to the anticancer activity of the drug. In conclusion, fascaplysin seems to be suitable for treatment of SCLC, based on high cytotoxic activity through multiple routes of action, affecting topoisomerase I, integrity of DNA and generation of ROS.

## 1. Introduction

Fascaplysin (12,13-Dihydro-13-oxopyrido[1,2-*a*:3,4-*b*′]diindol-5-ium chloride), a red bis-indole alkaloid of the marine sponge *Fascaplysinopsis* Bergquist sp., was first isolated in 1988, by Roll *et al.* [1]. This compound exhibited a broad range of activities including antibacterial, antifungal, antiviral, antimalarial, antiangiogenic and antiproliferative activity against numerous cancer cell lines [2,3,4]. Fascaplysin also showed DNA-intercalating capability with binding mode and affinity constants comparable to those of other typical DNA intercalators [5]. Additionally, significantly weaker non-intercalative DNA interactions were observed at high drug concentrations, pointing to its mechanism of biological activity via interference with genetic material. Furthermore, fascaplysin showed promising specific cyclin-dependent kinase 4 (CDK4) inhibitory activity with IC_50_ of 0.35 μM and it correspondingly blocked the growth of various cancer cells at the G0/1 phase of cell cycle [6,7]. Low activity was observed against other CDKs with IC_50_ of >100 μM for CDK1, >50 μM for CDK2, as well as 20 μM for CDK5. Recently, Shafiq and co-workers confirmed the specific effect of this compound on CDK4, which is known to play a key role in cell cycle control and is an important target for anticancer drugs [8]. Fascaplysin was reported to show cytotoxicity toward a panel of 60 cancer cell lines (IC_50_ values 0.6–4 μM), although testing was actually restricted to 36/60 of these cell lines [3]. The NCI60 panel misses small cell lung cancer (SCLC) cell lines altogether, a tumor entity that accounts for a significant fraction of lung cancer deaths [9]. 

A range of studies reported the anticancer activities of fascaplysin in cell lines *in vitro* and in experimental animal models. Fascaplysin did not provoke G1 phase arrest in HeLa cells although it led to downregulation of CDK4, cyclin D1 and CDK4-specific Ser795 retinoblastoma phosphorylation [10]. The molecular mechanism of fascaplysin-induced apoptosis was characterized as activation of caspase-3, -8, and -9, cleavage of Bid, release of cytochrome c into cytosol and downregulation of the level of Bcl-2. Fascaplysin can block VEGF, inhibit proliferation and induce apoptosis of human umbilical vein endothelial cells (HUVECs) [11,12]. The results showed that G1 cell cycle arrest was induced by 2.6 μM fascaplysin in a time-dependent manner, and HUVECs exhibited more chemosensitivity than hepatocarcinoma cells BeL-7402 and Hela cells. Apoptosis of HUVEC cells was induced by 1.3 μM fascaplysin and this response was further confirmed by the detection of active caspase-3, indicating involvement of a mitochondrial pathway. Microarray analysis show that the TNF and TNF receptor superfamily in HUVECs and BEL-7402 were significantly regulated by fascaplysin and this tumor necrosis-related apoptosis-inducing ligand-(TRAIL)-induced apoptosis resulted in activation of caspases 3 and 9 and decreases in Bid [13]. 

Fascaplysin was tested in a murine sarcoma S180 experimental animal model [14]. Treatment of the mice suppressed tumor growth significantly. Tumor sections showed hallmarks of apoptosis and the decreased expression of proliferating cell nuclear antigen (PCNA) and CD31 indicated cytostasis and antiangiogenesis. In another study involving fascaplysin, HCT-116 colon cancer cells were injected subcutaneously into severe combined immunodeficiency (SCID) mice [15]. At a tumor size of 250 mm^3^, mice received 4 mg/kg fascaplysin daily for five days. No toxicity was observed over the subsequent 30 days. At day 15, tumor size of the treated group was approximately 60% less than that of untreated control mice. Thus, even at this less than optimal dose, because a maxiumum tolerated dose (MTD) could not be obtained for fascaplysin, a therapeutic effect was observed.

In conclusion, cell line screening of the anticancer activity of fascaplysin is not complete and the mechanism inducing cell death in response to this drug, which may comprise different molecular mechanisms, is not clear. Interaction with other chemotherapeutic drugs to detect possible synergism was not described so far. Thus, in the present study, we screened SCLC cell lines, investigated cell cycle and cytotoxic effects of fascaplysin and used different drug combinations to screen putative synergistic action with established chemotherapeutics. In particular, fascaplysin was combined with camptothecins (CPTs) since we have reported enhancement of the cytotoxicity of the CPT analog topotecan against SCLC cell lines employing CDK4 inhibitors such as roscovitine and olomoucine [16]. 

**Figure 1:**
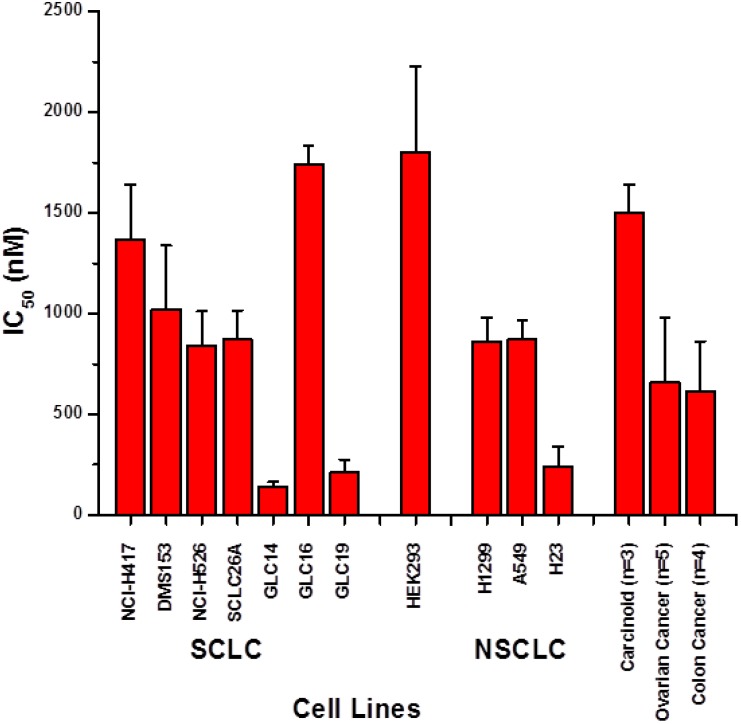
Screening of fascaplysin toxicity against small cell lung cancer (SCLC), non-small cell lung cancer (NSCLC), HEK293 and unrelated cell lines (mean values ± SD; *n* = 3).

## 2. Results and Discussion

### 2.1. Screening of Cytotoxic Activity against Lung Cancer Cell Lines

The cytotoxic activity of fascaplysin was assessed using a panel of SCLC cell lines using MTT assays (Figure 1). IC_50_ values measured ranged from 134 to 1740 nM fascaplysin. GLC14, 16 and 19 comprise a series of SCLC cell lines obtained from a single patient prior to chemotherapy and after first-line and second-line therapy, respectively. Whereas SCLC26A is an untreated chemosensitive SCLC cell line with IC_50_ values (μM ± SD) for cisplatin of 1.2 ± 0.6 and for etoposide of 0.5 ± 0.2, respectively, NCI-H417 and DMS153 are chemoresistant with IC_50_ values of 12.0 ± 2.8/3.3 ± 0.9 for cisplatin and 15.1 ± 2.2/12.9 ± 0.5 for etoposide, respectively. Therefore, sensitivity for fascaplysine differs by a factor of 1.2–1.6 between SCLC26A and DMS153/NCI-H417, but cisplatin and etoposide sensitivities differ 2.75–10 fold and 25.8–30 fold, respectively. These IC_50_ values observed did not exceed the corresponding value for the HEK293 cell line, representing normal kidney epithelial tissue. Fascaplysin IC_50_ values measured in SCLC cell lines are similar to the two chemoresistant NSCLC cell lines H1299 and A549 and the chemosensitive H23 cell line, respectively. Two pulmonary carcinoid cell lines showed sensitivities in the range of the SCLC cell lines and mean IC_50_ values for a panel of seven ovarian (PA-1, CaOV3, OV90, TUVGH211, A2780, A2780ADR) and five colon cancer (Colo205, HCT116, HCT116 p53 knockout, Colo320DM and HT29) cell lines indicated high chemosensitivity for fascaplysin.

**Figure 2:**
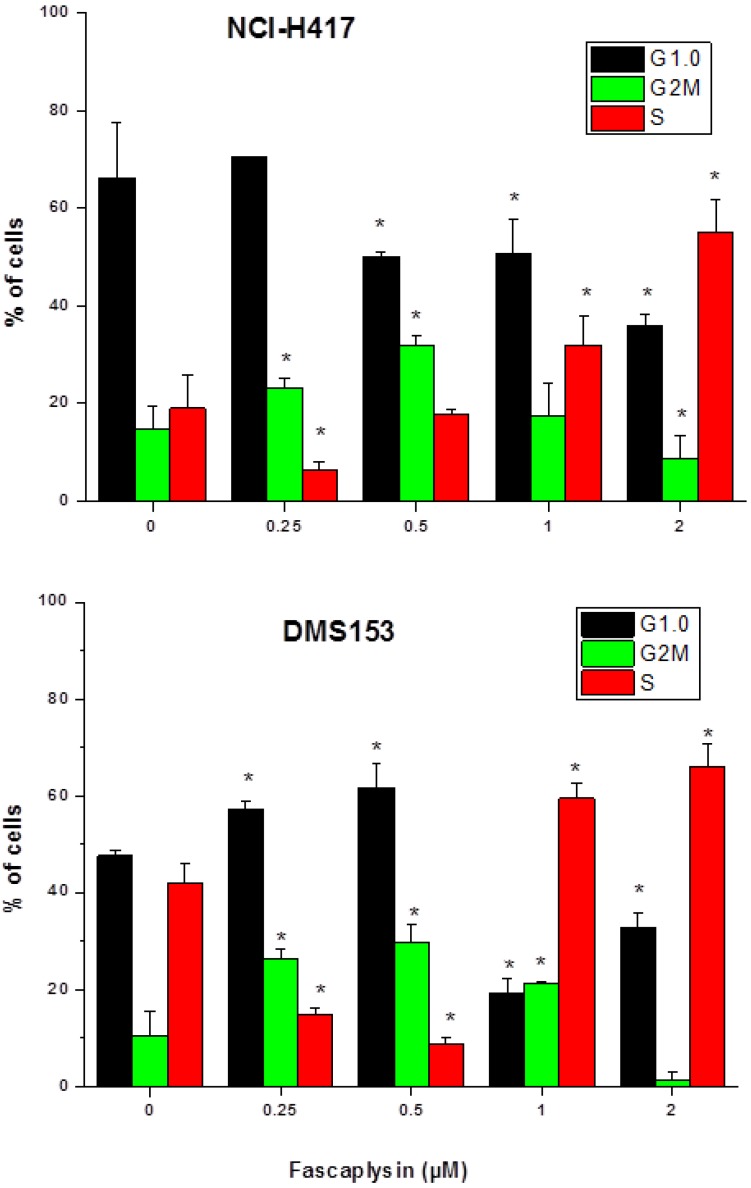
Cell cycle distribution of fascaplysin-treated NCI-H417 and DMS153 SCLC cells (mean ± SD; *n* = 3).

### 2.2. Effects of Fascaplysin on Cell Cycle Distribution of NCI-H417 and DMS153 Cells

For the assessment of effects of fascaplysin on cell cycle distribution in SCLC cells, NCI-H417 and DMS153 cells were treated with 0.25–2 μM of the compound and propidium iodide-stained cells analyzed in flow cytometry (Figure 2). The two lower concentrations of fascaplysin applied (0.25 and 0.5 μM) caused accumulation of cells in G2M and G1/0 phases, whereas the higher concentrations (0.5 and 1 μM) arrested cells preferentially in S-phase. 

**Figure 3:**
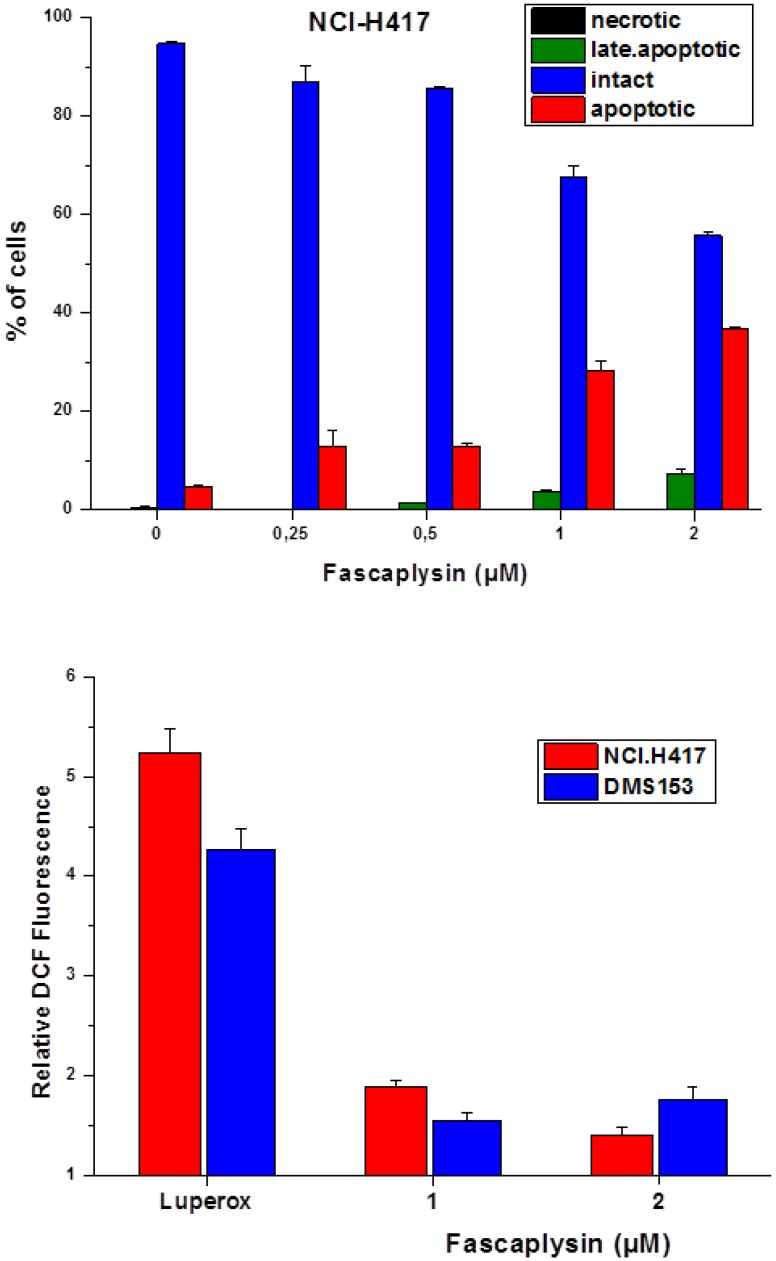
AnnexinV-propidium iodide staining of fascaplysin-induced apoptotic cell death in NCI-H417 cells (**A**) and effects of fascaplysin on intracellular ROS levels as detected by dichlorodihydrofluorescein (DCF) fluorescence in flow cytometry (**B**; mean ± SD; *n* = 3). For the apoptosis assay all differences to the control are significant, except for necrotic cells and late apoptotic cells at 0.25 nMfascaplysin. In the case of the ROS assay, all values are significantly different from those of untreated cells and values were calculated relative to the DCF fluorescence signal of untreated control cells.

### 2.3. Fascaplysin-Induced Apoptosis and Generation of ROS in SCLC Lines

Fascaplysin-induced apoptotic cell death was investigated in NCI-H417 cells using annexinV-propidium iodide double staining and flow cytometry (Figure 3A). Treatment of the cells with increasing concentrations of fascaplysin (0.25–2 μM) for four days resulted in the appearance of apoptotic and a small fraction of late apoptotic/necrotic tumor cells. 

Fascaplysin-induced generation of reactive oxygen species (ROS) in NCI-H417 and DMS153 cells was tested using fluorescence detection with the DCF dye (Figure 3B). A stabilized slow-release source of ROS, namely Di-tert-butyl peroxide/Luperox was included as positive control. Fascaplysin in concentrations of 1–2 μM was found to generate a limited but significant amount of ROS in both SCLC cell lines. *N*-acetyl-l-cysteine (NAC) is suitable for inactivating ROS and, accordingly, inclusion of 2.5–5 mM NAC in NCI-H417 fascaplysin cytotoxicity tests resulted in a twofold reduction of cell death (data not shown).

### 2.4. Synergistic Interaction of Fascaplysin and Camptothecins in NCI-H417 Cells

**Figure 4:**
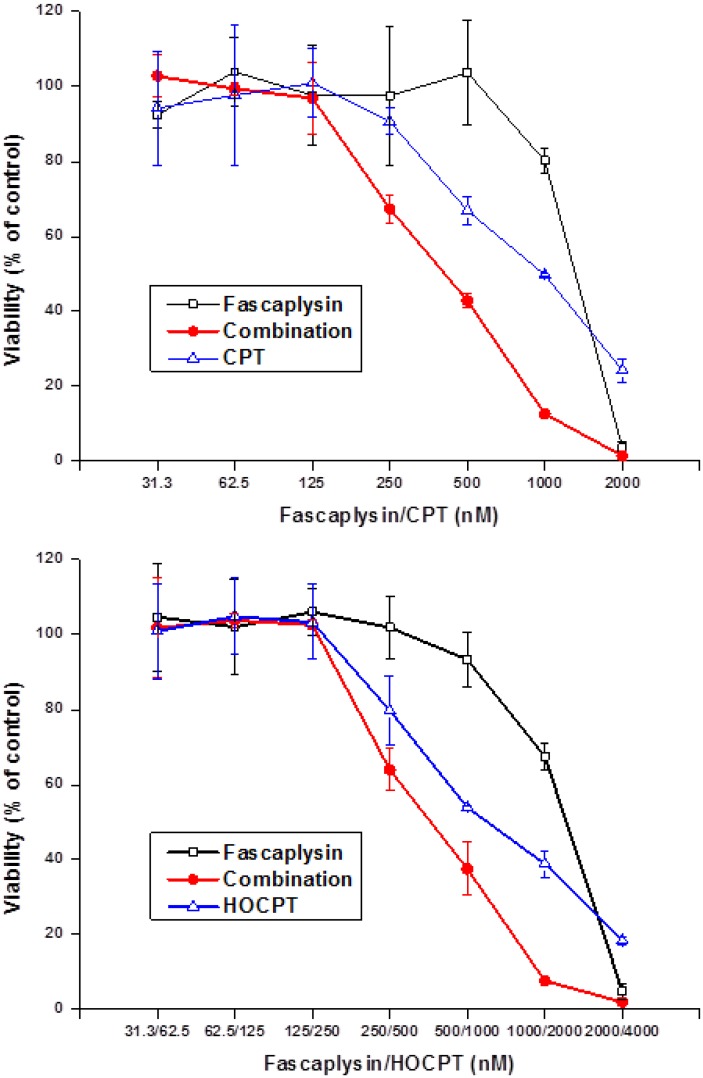
Combination of fascaplysin (initial concentration 2000 nM) with camptothecin (CPT, initial concentration 2000 nM; upper panel) or 10-hydroxy-camptothecin (HOCPT, initial concentration 4000 nM; lower panel) cytotoxicity assays using NCI-H417 cells (mean ± SD, *n* = 3).

Fascaplysin was tested for a possible interaction with camptothecin (CPT) and 10-hydroxycamptothecin (HOCPT) in MTT assays using chemoresistant NCI-H417 SCLC cells (Figure 4). Fascaplysin synergistically enhanced both CPT- and HOCPT-induced tumor cell death at specific drug concentrations. Combination indices (CI) were calculated using the CompuSyn software and yielded 0.73 and 0.56 for CPT concentrations of 1000 and 500 nM and 0.82 and 0.95 for HOCPT concentrations of 2000 and 1000 nM, respectively. A similar albeit weaker synergistic interaction was detected with the camptothecin analog, topotecan (data not shown). A range of other chemotherapeutic drugs comprising cisplatin, carboplatin and etoposide revealed no interaction with fascaplysin. Additionally, the P-glycoprotein (ABCB1) inhibitor PSC833 (1 and 2.5 μM) exhibited no effect on fascaplysin cytotoxicity against DMS153 cells. No interaction or minor antagonism was detected using combinations of fascaplysin with doxorubicin, etoposide, vinblastine, oxaliplatin, gemcitabine, docetaxel, mitomycin and cytarabin (data not shown).

### 2.5. Effects of the BYK 204165 PARP Inhibitor on Fascaplysin-Induced Cell Death in SCLC Cell Lines

Cytotoxicity assays using a combination of fascaplysin with the BYK 204165 PARP1 inhibitor were performed using a panel of SCLC cell lines. Whereas in chemosensitive NCI-H526 cells 50 and 25 μM BYK204165, respectively, yielded a minor chemosensitizing effect (factor 0.66; IC_50_ in presence of fascaplysin plus BYK204165 inhibitor/IC_50_ value in presence of fascaplysin), all other cell lines (NCI-H417, DMS153, GLC14 and GLC19) used showed strong antagonism for this combination with 2.9 ± 1.13 (range 1.83–4.46) and 1.9 ± 0.51 (range 1.48–2.6) increased chemoresistance compared to fascaplysin alone. 

### 2.6. Discussion

Fascaplysin was demonstrated to show cytotoxicity towards a large panel of cell lines in low μM concentrations. Similar anticancer activity was found in the present study against SCLC cell lines, yielding IC_50_ values comparable to those found in melanoma, colon cancer and ovarian cancer cell lines, among others. This compound exhibits high cytotoxicity independently of tissue origin and chemoresistance to unrelated drugs with few exceptions. Of the GLC14/16/19 series of SCLC cell lines, stemming from a single patient before and during two cycles of therapy failure, the GLC16 cells obtained after initial CHOP therapy showed highest resistance [17]. Likewise the H1299 and A549 NSCLC cell lines established from pretreated patients exhibited higher chemoresistance than H23, derived from tissue of an untreated patient [18,19]. However, the IC_50_ value obtained for the HEK293 epithelial kidney cell line exceeded those measured for most tumor cell lines, indicating a favorable toxicity profile. For further experiments, NCI-N417, a variant SCLC cell line known to be more aggressive and refractory to treatment although derived from a female patient with no prior treatment and DMS153, a line established from metastatic cells of a SCLC patient after therapy with cytoxan and methotrexate, were selected as chemoresistant cell lines [20,21]. 

As highly selective CDK4 inhibitor fascaplysin is expected to induce cell cycle arrest in G1/0 [6,7]. The activity of CDK-4 is restricted to the G1-S phases and is regulated by the attachment of the cyclin D and the endogenous CDK inhibitor p16INK4a. Both CDK4 and CDK6 encode cyclin-dependent serine-threonine kinases that, complex with D-type cyclines to phosphorylate the RB tumor suppressor protein, in turn resulting in transcription of genes required for G1-S phase cell cycle progression [22]. Fascaplysin-induced cell cycle perturbations in NCI-H417 and DMS153 are not restricted to G1/0 and seem to be insufficient to trigger apoptosis via inhibition of CDK4. In SCLC the tumor suppressor circuit comprising p16INK4, which specifically binds and inhibits CDK4/6, and RB is inactivated [23,24]. A minor fraction of SCLC tumors have absent p16INK4 protein and wildtype RB expression, whereas the majority of tumors which possess detectable levels of p16INK4 protein hold absent or mutant RB. Fascaplysin-induced G1 arrest is dependent on intact Rb protein. Absence of Rb protein was published for DMS153 cells [25] and the same result was found here for NCI-H417 cells with help of the Poly6146 rabbit antibody (Biolegend, San Diego, CA, USA) and paraformaldehyde-fixed cells (data not shown). Additionally, both SCLC cell lines, NCI-H417 and DMS153, feature mutated p53 [26,27].

Fascaplysin induces apoptotic cell death in NCI-H417 cells dose-dependently and the generation of ROS seems to support this cytotoxicity since addition of *N*-acetyl-l-cysteine (NAC) to MTT tests partially reverse the cytotoxic effect of this compound. NAC can interact directly with reactive oxygen species (ROS) because it is a scavenger of oxygen free radicals [28,29]. Drug combinations comprising fascaplysin and a range of chemotherapeutics yielded synergism with CPT and HOCPT solely. This finding may be explained either as fascaplysin acting as CDK4 inhibitor or, more likely in the two SCLC lines studied, as interfering with the camptothecin-toposiomerase I-DNA cleavable complex. We have described the chemosensitizing effects of CDK4 inhibitors in conjunction with CPTs recently [16]. In limited support of the role of fascaplysin as CDK4 inhibitor we found antagonism with a CDK4 inhibitor (EMD/Millipore, Darmstadt, Germany), no interaction with roscovitine/olomoucine and synergism with the pan-CDK inhibitor flavopiridol in MTT cytotoxicity assays (data not shown). 

In order to further check possible DNA damage and repair we applied the isoquinolindione BYK204165 selective PARP1 inhibitor to fascaplysin cytotoxicity tests against different SCLC cell lines [30]. The basal activity of PARP1 is very low, but is stimulated markedly under cellular stress/oxidative damage. Under conditions of low to moderate DNA damage, PARP provides cytoprotection, but in case of extended damage and high PARP activation cells are eliminated by apoptosis [31,32]. Consequently, depending on the circumstances, inhibitors of PARP either enhance the cytotoxicity of drugs or provide protection [33]. In the presence of a PARPi, PARP-1 binds DNA strand breaks but cannot produce poly(ADP-ribose) polymers and since DNA binding is persistent repair is impaired. The cytotoxicity of fascaplysin is impaired by the PARP1 inhibitor in the SCLC cell lines tested, except the chemosensitive NCI-H526 cells. This effect may be partially due to the fascaplysin-induced cell cycle arrest in S-phase. The increased resistance of the SCLC cell lines in presence of the PARP1 inhibitor point to an inhibition of the fascaplysin-triggered induction of cell death, possibly by prevention of NAD^+^resynthesis and ATP depletion and/or translocation of apoptosis-inducing factor (AIF) from the mitochondria to the nucleus [34].

## 3. Experimental Section

### 3.1. Reagents and Cell Lines

Stock solutions of all compounds were prepared in DMSO. All other chemicals were purchased from Sigma-Aldrich (St. Louis, MO, USA), except indicated otherwise. The BYK 204165 PARP1 inhibitor was obtained from Tocris Bioscience (Bristol, UK). Cell lines were obtained from ATCC (Rockville, MD, USA), except DMS153 cells from ECACC (Porton Down, Salisbury, UK), the GLC14/16/19 series from Dr. Nina Pedersen from the Department of Radiation Biology, The Finsen Centre, National University Hospital, Copenhagen, Denmark and H1299 and H23 from the University of Graz. The SCLC26A line was established from a pleural effusion of an untreated patient with SCLC at our institution. Cells were grown in RPMI-1640 bicarbonate medium (Seromed, Berlin, Germany) supplemented with 10% fetal bovine serum (Seromed, Berlin, Germany), 4 mM glutamine and antibiotics (10× stock formulated to contain ~5000 units penicillin, 5 mg streptomycin and 10 mg neomycin/mL) under tissue culture conditions (37 °C, 5% CO_2_, 95% humidity) and checked for mycoplasma contamination (Mycoplasma PCR ELISA, Roche Diagnostics, Vienna, Austria).

### 3.2. Chemosensitivity Assay

1 × 10^4^ cells in 100 μL medium per well were distributed in 96-well microtiter plates (Greiner, Kremsmuenster, Austria) and the test compound added in another 100 μL. Drugs and solute controls were serially diluted in twofold steps in triplicate. The microtiter plates were incubated under tissue culture conditions for four days and cell viability was measured using a modified MTT (3-(4,5-dimethylthiazol-2-yl)-2,5-diphenyl-tetrazolium bromide) assay (EZ4U, Biomedica, Vienna, Austria). Optical density was measured using a microplate reader at 450 nm with an empty well as reference. Values obtained from control wells containing cells and media alone were set to 100% proliferation. For tests of synergy, compounds were diluted individually and in combination, using the same (CPT) or double (HOCPT) initial concentrations. The synergistic effect of drug combinations was assessed using CompuSyn (V 1.03; CompuSyn Inc., Paramus, NJ, USA), a software program based on the calculations for synergism developed by Chou *et al.* [35]. Combinations with a combination index (CI) <1 were considered synergistic.

### 3.3. Measurement of Cell Cycle Distribution

1 × 10^6^ cells per well were incubated with the respective compound in six-well plates for three days. Harvested cells were washed with PBS and fixed with 70% ethanol at −20 °C for 30 min, washed again, transferred into staining solution (20 μg/mL propidium iodide (PI), 5 μg/mL ribonuclease A, 0.05% Nonidet P40 in PBS) and incubated at room temperature overnight. Washed cells were analyzed by acquisition of 1 × 10^4^ cells by flow cytometry (Cytomics FC500, Beckman Coulter, Krefeld, Germany) at excitation and emission wavelengths of 488 and 675 nm, respectively. The proportion of subG1 (apoptotic) cells was obtained from the logarithmic PI histograms, and percentages of cells in cell cycle phases G1/0 (resting), S (DNA synthesis) and G2M (mitotic) were calculated from linear PI histograms using MultiCycle AV software (Phoenix Flow Systems, San Diego, CA, USA). Experiments were done in duplicate.

### 3.4. Detection of Reactive Oxygen Species (ROS)

1 × 10^6^ washed cells were preincubated with 5 μg/mL 2′,7′-dichlorodihydrofluorescein diacetate (DCF), a chemically reduced, acetylated form of fluorescein, in phosphate buffered saline (PBS) at 37 °C for 15 min. This indicator is readily converted to a green-fluorescent form following removal of the acetate groups by intracellular esterases and oxidation by ROS. Cells which were washed again were then treated with fascaplysin under tissue culture conditions at 37 °C for 4 h. Identical treatment of the cells with Luperox^®^ TBH70X, Di-tert-butyl peroxide solution, gave the positive controls. Samples were subsequently analyzed by flow cytometry.

### 3.5. Statistical Analysis

Statistical differences were calculated using student’s paired *t*-test at significance levels of *p* < 0.05 (indicated in the figures by an asterisk).

## 4. Conclusions

Fascaplysin exhibits cytotoxicity against chemoresistant SCLC cell lines via several contributing pathways comprising, among others, generation of ROS and cellular mechanisms affecting topoisomerase I-activity and activation of PARP, possibly linked to its interaction with DNA in absence of a functional CDK4-RB1 axis in SCLC cell lines. Synergistic interaction with camptothecins is of special interest for second-line treatment of SCLC relying on the camptothecin analog topotecan.
